# Internet Treatment for Generalized Anxiety Disorder: A Randomized Controlled Trial Comparing Clinician vs. Technician Assistance

**DOI:** 10.1371/journal.pone.0010942

**Published:** 2010-06-03

**Authors:** Emma Robinson, Nickolai Titov, Gavin Andrews, Karen McIntyre, Genevieve Schwencke, Karen Solley

**Affiliations:** 1 Clinical Research Unit for Anxiety and Depression, St Vincent's Hospital, Sydney, New South Wales, Australia; 2 Clinical Research Unit for Anxiety and Depression, School of Psychiatry, University of New South Wales at St Vincent's Hospital, Sydney, New South Wales, Australia; University of Granada, Spain

## Abstract

**Background:**

Internet-based cognitive behavioural therapy (iCBT) for generalized anxiety disorder (GAD) has been shown to be effective when guided by a clinician. The present study sought to replicate this finding, and determine whether support from a technician is as effective as guidance from a clinician.

**Method:**

Randomized controlled non-inferiority trial comparing three groups: Clinician-assisted vs. technician-assisted vs. delayed treatment. Community-based volunteers applied to the VirtualClinic (www.virtualclinic.org.au) research program and 150 participants with GAD were randomized. Participants in the clinician- and technician-assisted groups received access to an iCBT program for GAD comprising six online lessons, weekly homework assignments, and weekly supportive contact over a treatment period of 10 weeks. Participants in the clinician-assisted group also received access to a moderated online discussion forum. The main outcome measures were the Penn State Worry Questionnaire (PSWQ) and the Generalized Anxiety Disorder-7 Item (GAD-7). Completion rates were high, and both treatment groups reduced scores on the PSWQ (p<0.001) and GAD-7 (p<0.001) compared to the delayed treatment group, but did not differ from each other. Within group effect sizes on the PSWQ were 1.16 and 1.07 for the clinician- and technician-assisted groups, respectively, and on the GAD-7 were 1.55 and 1.73, respectively. At 3 month follow-up participants in both treatment groups had sustained the gains made at post-treatment. Participants in the clinician-assisted group had made further gains on the PSWQ. Approximately 81 minutes of clinician time and 75 minutes of technician time were required per participant during the 10 week treatment program.

**Conclusions:**

Both clinician- and technician-assisted treatment resulted in large effect sizes and clinically significant improvements comparable to those associated with face-to-face treatment, while a delayed treatment/control group did not improve. These results provide support for large scale trials to determine the clinical effectiveness and acceptability of technician-assisted iCBT programs for GAD. This form of treatment has potential to increase the capacity of existing mental health services.

**Trial Registration:**

Australian New Zealand Clinical Trials Registry ACTRN12609000563268

## Introduction

Generalized anxiety disorder (GAD) is a common anxiety disorder characterized by chronic, excessive, and uncontrollable worry. The 12-month prevalence of GAD in Australia and the US is 2.7% and 3.1%, respectively [1], [2]. GAD commonly co-occurs with other anxiety disorders and/or depression and is highly disabling, resulting in levels of disability comparable to depression [3], [4]. Although people with GAD frequently utilise health care facilities they often report somatic rather than psychological symptoms, making diagnosis difficult [5].

GAD can be treated effectively with cognitive behavioural therapies (CBT) [6]–[8], but numerous barriers to treatment exist, including the direct and indirect costs of treatment, the limited availability of mental health professionals, stigma, and the difficulty of patients attending treatment during office hours [9], [10]. One promising strategy for reducing these barriers involves Internet-based CBT (iCBT) programs. iCBT programs result in clinically significant improvements in patients with depression [11]–[14], panic disorder [15]–[18], social phobia [19]–[26], with encouraging results recently reported from the first iCBT program for treating GAD [27]. In that study, treatment group participants obtained clinically significant reductions in severity of GAD symptoms relative to a control group.

The successful use of a non-clinician (technician) to administer iCBT, without compromising clinical outcomes, has been reported in iCBT programs for depression and social phobia [14], [25], [26]. The use of technicians to oversee administration of iCBT programs has considerable implications for the cost-effectiveness of iCBT. An important question is whether similar effects could be obtained from iCBT for GAD.

The present CONSORT-Revised compliant randomized controlled trial (RCT) [28] had two aims: To replicate the recent finding that people with a DSM-IV [29] diagnosis of GAD could be treated using the Worry program [27], a diagnosis-specific iCBT program developed to treat GAD, and; to examine the relative clinical efficacy and acceptability of clinician- and technician-assisted iCBT using the Worry program [27]. We tested four hypothesises: Firstly, that clinician-assisted (CA) treatment would be efficacious; secondly, that participants in a technician-assisted (TA) group would show similar clinical improvements on measures of GAD, depression and disability as the clinician-assisted (CA) group; thirdly, that both treatment groups would have better outcomes than a delayed treatment (control) group; and finally; improvements would be sustained at follow-up.

## Methods

The protocol for this trial and supporting CONSORT checklist are available as supporting information; see CONSORT Checklist S1 and Protocol S1.

### Ethics

This study was approved by the St Vincent's Hospital Human Research Ethics Committee and by the University of New South Wales Human Research Ethics Committee. Written informed consent was obtained from all participants.

### Participants

Participants were recruited from July to September 2009 via a website (www.virtualclinic.org.au) providing information about common mental disorders including GAD, and a link to apply online to join a research treatment program. Participants first applied online, completing several screening questionnaires about the presence and severity of symptoms of anxiety and depression, including the Patient Health Questionnaire - 9 Item (PHQ-9) [30], the Generalized Anxiety Disorder 7-Item Scale (GAD-7) [31] and the Social Phobia Screening Questionnaire (SPSQ) [32]. Questions were also asked to determine demographic details of participants (see Table 1).

**Table 1:** Demographic description of participants.

		*Technician-Assisted*		*Clinician-Assisted*		*Control Group*		*Total*	
		*(n = 50)*		*(n = 46-47)* *		*(n = 47–48)* *		*(n = 144–145)* *	
Variable	Sub-variable	*n*	%	*n*	%	*n*	%	*n*	%
Gender	Male	19	38.0	13	27.6	14	29.1	46	31.7
	Female	31	62.0	34	72.3	34	70.8	99	68.3
Age	Mean Age (SD)	44.16 (12.44)		45.57 (13.14)		51.23 (11.61)		46.96 (12.70)	
	Range	18–68		25–68		21–80		18–80	
Marital Status	Single/Never Married	17	34.0	9	19.1	5	10.4	31	21.3
	Married/De Facto	26	52.0	37	78.7	30	62.5	93	64.3
	Separated/Divorced	7	14.0	1	2.1	13	27.0	21	14.4
Education	High school	6	12.0	6	12.8	6	12.5	18	12.4
	Tertiary	33	66.0	32	68.1	34	70.8	99	68.3
	Other Certificate	11	22.0	7	14.8	8	16.7	26	17.9
	None	0	0.0	2	4.3	0	0	2	1.4
Employment Status	Part time/student	16	32.0	12	26.1	19	39.6	47	32.4
	Full time	25	50.0	19	41.3	19	39.6	63	43.4
	Unemployed, retired or disabled	9	18.0	15	32.6	10	20.8	34	23.4
Previously Discussed Symptoms with Health Professional		40	80.0	34	72.3	35	74.5	109	75.2
Taking Medication		17	34.0	16	34.0	14	29.2	47	32.4
Hours/Week of Internet use.						missing data (n = 1)	n = 48	N = 145	
	0–10	24	48.0	23	48.9	30	63.8	77	53.1
	11+	26	52.0	24	51.1	17	36.2	67	46.2
Confidence using computers and Internet	Very Confident	29	58.0	24	51.1	26	54.2	79	54.5
	Confident	15	30.0	15	31.9	12	25	42	29.0
	Average	6	12.0	6	12.8	6	12.5	18	12.4
	Mildly Confident	0	0	2	4.3	2	4.2	4	2.8
	Not Confident	0	0	0	0	2	4.2	2	1.4

Note: *Missing data.

Exclusions were (i) not a resident of Australia; (ii) less than 18 years of age; (iii) no regular access to a computer, the Internet, and use of a printer; (iv) currently participating in CBT; (v) using illicit drugs or consuming more than three standard drinks/day; (vi) experience of a psychotic mental illness (schizophrenia or bipolar disorder) or current severe symptoms of depression (defined as a total score >23 or responding >2 to Question 9 (suicidal ideation) on the PHQ-9; and (vii) if taking medication, had been taking the same dose for less than 1 month or intending to change that dose during the course of the program. Excluded applicants immediately received an on-screen message and email thanking them for their application, and encouraging them to discuss their symptoms with their physician.

Participants who passed the screening phase were telephoned for a diagnostic interview using the Mini International Neuropsychiatric Interview Version 5.0.0 (MINI) [33] to determine whether they met DSM-IV criteria for GAD. Participants who satisfied all criteria were informed of the study design and invited to return a completed consent form by email. The study was approved by the Human Research Ethics Committees of St Vincent's Hospital, Sydney, and the University of New South Wales.

### Interventions

Treatment groups received access to the Worry program, an iCBT program with demonstrated efficacy at reducing symptoms of GAD [27]. The Worry program consists of six online lessons, printable summary and homework assignments, automatic emails, and additional resource documents. The six online lessons represent best practice principles used in CBT programs for GAD including cognitive therapy, challenging meta-beliefs about worry, graded exposure, challenging core beliefs, and relapse prevention. Part of the content of each lesson is presented in the form of an illustrated story about a woman with GAD who, with the help of a clinical psychologist, learns to gain mastery over her symptoms. Automatic emails are sent to congratulate participants for completing each lesson, to remind them to complete materials, and to notify them of new resources. As people progress through each lesson they have access to additional written documents providing supplementary information about techniques such as managing sleep problems, assertiveness and problem solving skills, managing low mood, panic, and other common comorbid symptoms. They are also provided with access to vignettes written by previous participants about their own experiences in the Worry program of managing GAD. Participants are expected to complete the homework tasks prior to completing the next lesson, and to complete all lessons within 10 weeks.

All participants in the treatment groups began the 10-week treatment program at the same time. Participants were advised to complete one lesson every 7–10 days and to complete the six lessons within 10 weeks of starting. All participants received automatic emails informing them when a lesson was to be completed, and reminder emails if they had not completed a lesson within 7 days of notification.

Three staff conducted the study, with supervision from NT. The technician (KM) was employed in an administrative role as a Clinic Manager, Anxiety Disorders Clinic, Mental Health Service, St Vincent's Hospital, Sydney. She reported no prior experience with research programs, no qualifications in health care or counseling, and had no clinical duties in her usual role. The clinician (ER) was a qualified and registered clinical psychologist, employed at the same unit as KM. The third staff member (KS) was a research assistant, who provided administrative support to the technician and clinician.

#### Technician-Assisted Treatment

During treatment the technician provided TA group participants with weekly email or telephone contact. The technician was given a guideline script which identified the topics covered in each lesson of the program and activities participants should be encouraged to practice for each lesson. The technician was instructed to contact each TA group participant weekly to provide encouragement and support, and where possible to respond to participants' general questions by referring them to the materials in the Worry program. The technician was not permitted to provide clinical advice. The technician received supervision from the clinician and was instructed to inform the clinician of any perceived deterioration in the participants' mental health status, or of any concerns about participants' wellbeing. While conducting this research the technician maintained her full-time role as a Clinic Manager.

#### Clinician-Assisted Treatment

CA group participants had weekly email or telephone contact with the clinician and access to an online discussion forum where they could post questions to the clinician about the program content. Information posted on the discussion forum could be read by other participants in the CA group. The clinician was provided with the same guideline script as the technician but was also instructed to answer participants' questions via forum, email, or telephone. The clinician was instructed to actively engage with each participant in treatment including goal setting, problem solving, and discussion of strategies for overcoming hurdles to progress. Because of the clinical nature of messages on the forum, the TA group did not have access to a forum. The clinician and technician were instructed to try to spend no more than 10 minutes in contact with each participant per week. The total time required and nature of all contacts with participants during treatment was recorded.

#### Control Group

Control group participants received no treatment for 11 weeks and then received the clinician-assisted program described above, beginning treatment one week after the intervention groups completed the Worry program.

### Objectives

This study was a 3 group randomized controlled non-inferiority trial to determine whether technician-assisted iCBT was equivalent to clinician-assisted iCBT but superior to delayed treatment (control).

### Primary and Secondary Outcomes

#### Outcomes

One week prior to beginning the trial participants completed the following questionnaires online: The Penn State Worry Questionnaire (PSWQ) [34]; the GAD-7; the Patient Health Questionnaire 9-Item (PHQ-9); the Kessler 10 (K-10) [35]; the Sheehan Disability Scales (SDS) [36]; and the Credibility/Expectancy Questionnaire (CEQ) [37], [38]. The PSWQ is a 16-item measure with scores ranging from 16–80. The GAD-7 is a 7-item measure with scores ranging from 0–27. The PSWQ and GAD-7 are frequently used clinical and research measures of GAD. A score of 10 on the GAD-7 has been identified as providing an important threshold for identifying DSM-IV congruent GAD [31]. The PHQ-9 is a 9-item measure of depressive symptoms with scores ranging from 0–27. The K-10 is a 10-item measure of psychological distress with scores ranging from 10–50. The SDS is a 3-item measure of disability with scores ranging from 0 to 30 and the CEQ is a widely used measure of the expectancies or perception of treatment credibility.

The PSWQ, GAD-7, PHQ-9, K-10, SDS and a treatment satisfaction questionnaire (based on the CEQ) were re-administered one week post-treatment and at three-months post-treatment (follow-up), while the GAD-7 and PHQ-9 was also administered mid-treatment (at week 5). All of these measures are considered reliable, valid, and appropriate for clinical and research purposes, with recent research indicating that online administration of questionnaires results in acceptable reliability of responses [39], [40]. Changes in the PSWQ and GAD-7 were considered the primary outcome measures, while changes in the PHQ-9, K-10, SDS, and treatment satisfaction questionnaire were the secondary outcome measures. Results are reported at the end of treatment. Follow-up results were not available for the control group, who had started treatment by that time.

### Sample Size and Randomization

Power calculations were based on a non-inferiority trial design comparing parallel-groups. Alpha was set at 0.025, power at 90%, and the mean minimal reliable change index on the GAD-7 (based on earlier findings) and standard deviations for each group were expected to be equivalent (5 and 4, respectively). Using Table V from Julious [41], the minimum sample size for each group was identified as 39, but more were recruited to hedge against attrition.

**Figure 1:**
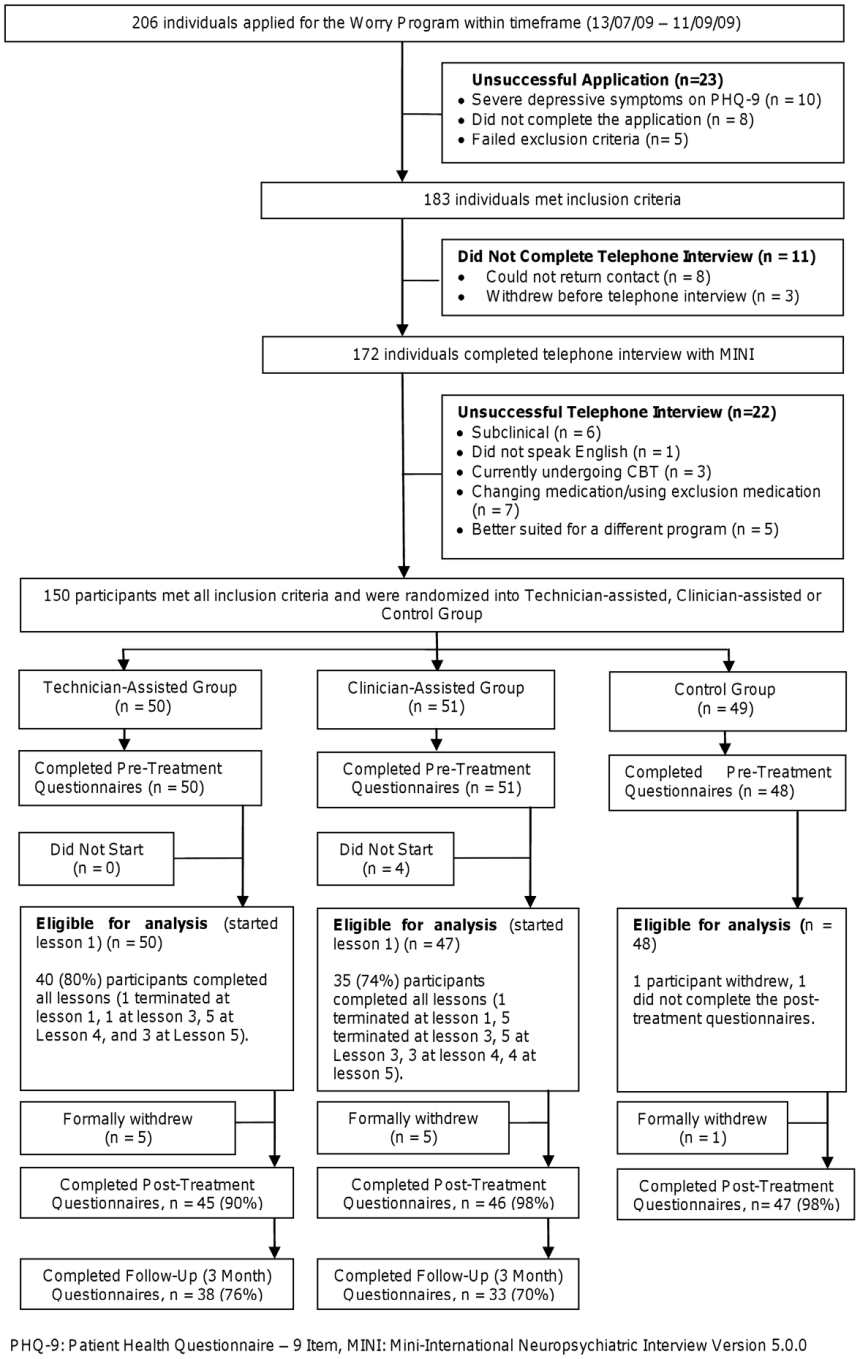
CONSORT-R participant flow chart.

The 150 people accepted into the program were randomised by NT via a true randomisation process (www.random.org) to either the CA (n = 51), TA (n = 50), or control groups (n = 49) (see Figure 1). Allocation preceded the diagnostic telephone call. Dependence on self-report measures precluded blinding.

### Statistical Analysis

Group differences in demographic data, pre-treatment measures, and pre-treatment expectations were analyzed with one-way analysis of variance (ANOVAs) and chi-square tests, followed by t-tests with Bonferonni corrected p values. Changes in participants' questionnaire scores from pre to post-treatment and from pre-treatment to follow-up were analyzed using repeated measures analyses of covariance (ANCOVAs). This approach is recommended as a robust and reliable statistical strategy for analyzing the results of RCTs [42], [43]. Changes in questionnaire scores between post-treatment and follow-up were analyzed using paired samples t-tests. Effect sizes (Cohen's d) were calculated both within- and between-groups, based on the pooled standard deviation.

All post-treatment and follow-up analyses adopt an intention-to-treat (ITT) design where missing data is replaced by the last observation carried forward (LOCF).

Two scores (credibility and expectancy) were derived from the CEQ as described in [37].

Three measures of clinical significance were employed. Pre-treatment and post-treatment GAD-7 scores were compared with optimum cut-offs for a probable diagnosis of GAD [44], to provide an index of *remission*. This was defined as the proportion of participants who initially scored above the optimum cut-off (GAD-7 total score of 10 or more) and subsequently scored below this cut-off. Secondly, an estimate of *recovery* was made by identifying the proportion of participants in each group who demonstrated a significant reduction in their symptoms (defined here, as a reduction of 50% of pre-treatment GAD-7 scores), as described in recent dissemination studies [45]. Thirdly, the percentage of participants in each group who met criteria for reliable change on the PSWQ was calculated. This was defined as the proportion of participants who met the criteria of statistically reliable change as described in Jacobson and Truax [46]. A reliable change index for the PSWQ was calculated separately for each of the three groups using their pre-treatment standard deviation, and a test-retest reliability coefficient of 0.93, as reported in [34].

## Results

### Participant flow

Two hundred and six individuals expressed interest in the study (Figure 1), 150 met the eligibility criteria and were randomized to one of the three groups. Fifty TA and 47 CA group participants completed the pre-treatment questionnaires and began Lesson 1 and are eligible for analysis along with 48 control group participants who completed the pre-treatment questionnaires. Five of the original 150 participants did not complete the pre-treatment questionnaires, and so are ineligible for analysis.

### Baseline Characteristics

Characteristics of the groups are presented in Table 1. There were no significant between-group differences in gender, education, employment, previous discussions of symptoms with a health professional, use of medication, weekly use of the internet, or confidence in using computers. Treatment groups were also equally chronic, with 71% of treatment group participants reporting onset of GAD before age 30 years. At pre-treatment, both treatment groups also rated the likely benefits of the Worry program as similar, although participants in the CA group rated the expectation of benefit as marginally greater than the TA group (*F_1, 91_* = 3.77, p<0.06).

There was a significant difference between groups in marital status (χ^2^ = 19.48, *df* = 4 p<0.001), but post-hoc tests removing the control group revealed no differences between the treatment groups. A one-way ANOVA revealed significant between-groups differences in age (*F_2, 142_* = 4.41, p<0.01), with Bonferronni corrected post-hoc t-tests revealing that participants in the control group (M = 51.23, SD = 11.61) were significantly older than TA group (M = 44.16, SD = 12.44) and CA group (M = 45.57, SD = 13.14) participants, with no differences between treatment groups. ANOVAs were also conducted to explore pre-treatment differences in symptom severity. No between group differences were found on the PSWQ, GAD-7, PHQ-9, K-10, or SDS (p>0.05).

#### Completion Rates

Forty (40/50, 80%) TA and 35/47 CA (74%) group participants completed all 6 lessons within the required time. Reasons for not completing all lessons were not collected. Post-treatment data was collected from 45 (90%) TA, 46 (98%) CA group members, and from 47/48 (98%) of control group participants. Follow-up data (3 months post-treatment) were collected from 38/50 (76%) TA and 33/47 (70%) CA group participants. In accordance with the ITT and LOCF paradigm, the pre-treatment scores of the participants who did not complete the post-treatment questionnaires were replicated as their post-treatment scores.

### Post-Treatment (11-week) Outcomes

#### Primary Outcomes

Univariate ANCOVAs on post-treatment PSWQ and GAD-7 scores, controlling for pre-treatment scores (see Table 2), revealed significant effects for PSWQ (*F_2, 141_* = 23.02, p<0.001) and GAD-7 (*F_2, 141_* = 27.04, p<0.001) scores. Post-hoc pairwise comparison of groups revealed no difference on either measure between treatment groups, but significant differences between the treatment groups and the control group (p<0.001). The effect of the differences in age between groups were explored by repeating these calculations and adding age as a covariate, but age was not significantly related after controlling for pre-treatment scores.

**Table 2:** Results of outcome measures: Means, standard deviations, confidence intervals and effect sizes (Cohen's *d*) for each group (intention to treat; last observation carried forward).

	Group		Pre	Post	Pre-post	Effect Sizes*			Follow-Up	Pre-Follow-Up	Effect Sizes*	
Outcome Measure		n	Mean (SD)	Mean (SD)	Mean Difference (95% CI)	Within Group	TA vs. CA	vs. Control	Mean (SD)	Mean Difference (95% CI)	Within	TA vs. CA
PSWQ	TA	50	63.12 (9.46)	52.28 (10.73)	10.84 (7.95–13.73)	1.07	0.07	1.06	52.52 (12.29)	10.60 (7.66–13.54)	0.97	0.34
	CA	47	64.02 (9.27)	51.45 (12.28)	12.57 (9.26–15.89)	1.16		1.06	48.26 (12.63)	15.77 (12.31–19.22)	1.42	
	Control	48	65.81 (10.24)	64.22 (11.81)	1.40 (−0.23–3.02)	0.14						
GAD-7	TA	50	11.90 (3.38)	6.02 (3.43)	5.88 (4.66–7.10)	1.73	0.11	1.25	6.26 (3.64)	5.64 (4.36–6.92)	1.61	0.16
	CA	47	12.45 (4.14)	5.55 (4.73)	6.89 (5.35–8.44)	1.55		1.05	5.55 (5.14)	6.89 (5.33–8.46)	1.48	
	Control	48	12.94 (4.07)	11.25 (4.70)	1.69 (0.62–2.76)	0.38						
PHQ-9	TA	50	12.08 (5.21)	6.28 (5.04)	5.80 (4.16–7.44)	1.13	0.13	0.91	6.00 (4.99)	6.08 (4.67–7.49)	1.19	0.05
	CA	47	11.40 (4.63)	5.62 (5.14)	5.79 (4.24–7.33)	1.18		1.02	5.72 (5.79)	5.68 (4.10–7.26)	1.08	
	Control	48	12.50 (4.73)	10.94 (5.25)	1.56 (0.39–2.73)	0.31						
K-10	TA	50	27.48 (6.76)	20.56 (6.86)	6.92 (5.12–8.72)	1.02	0.10	0.78	20.46 (7.38)	7.02 (5.10–8.94)	0.99	0.13
	CA	47	27.34 (7.29)	19.83 (7.75)	7.51 (5.62–9.40)	0.99		0.83	19.45 (8.49)	7.89 (5.78–10.01)	1.00	
	Control	48	27.35 (6.77)	25.94 (6.93)	1.42 (−0.09–2.92)	0.21						
SDS	TA	50	16.92 (7.44)	8.98 (7.87)	7.94 (5.81–10.07)	1.04	0.05	0.87	8.22 (7.89)	8.70 (6.57–10.83)	1.13	0.01
	CA	47	14.85 (7.72)	9.40 (9.37)	5.45 (3.41–7.48)	0.63		0.74	8.17 (9.63)	6.68 (4.41–8.96)	0.77	
	Control	48	15.08 (8.31)	15.75 (7.71)	−0.67 (−3.01–1.68)	0.08						

Pre: Pre treatment, Post: post-treatment; 4-month: 4 month follow-up. PSWQ: Penn State Worry Questionnaire; GAD-7: Generalized Anxiety Disorder 7-Item; PHQ-9 Patient Health Questionnaire 9-Item; K-10 Kessler 10-Item; SDS Sheehan Disability Scale; TA technician assisted; CA clinician assisted. CI Confidence Interval.

Note. *All effect sizes are absolute values.

#### Secondary Outcome

Univariate ANCOVAs conducted on the PHQ-9, K-10, and SDS post-treatment scores, while controlling for pre-treatment scores revealed significant effects over time for the PHQ-9 (*F_2, 141_* = 16.63, p<0.001), K-10 (*F_2, 141_* = 17.52, p<0.001), and SDS (*F_2, 141_* = 17.57, p<0.001) scores. Post-hoc pairwise comparison of groups revealed no difference on either measure between treatment groups, but significant differences between the treatment groups and the control group (p<0.001).

#### Effect Sizes

Within-group effect sizes on the PSWQ were 1.16 and 1.07 for the clinician- and technician-assisted groups, respectively, and on the GAD-7 were 1.55 and 1.73, respectively. Large (>0.80) within-group effect sizes (ESs) (Table 2) were found for both treatment groups on the PHQ-9 and K-10, and on the SDS for the TA group. Large ESs between each treatment group and the control group were found for most measures.

#### Clinical Significance: Remission, Recovery, and Reliable Clinical Change

At pre-treatment 37/50 (74%) of TA group, 32/47 (68%) of CA group, and 36/48 (75%) of control group participants had a GAD-7 score of 10 or more, indicating a diagnosis of GAD. At post-treatment (using the intention-to-treat and LOCF design), 8/50 (16%) of TA group, 8/47 (17%) of CA group, and 29/48 (60%) of the control group participants continued to have a GAD-7 score above 9. Based on the criteria for recovery (a reduction of pre-treatment GAD-7 scores of at least 50%) at post-treatment, 28/50 (56%) of TA group, 33/47 (70%) of CA group and 4/48 (10%) of control group participants were classified as recovered. Based on the criteria for reliable clinical change (statistically reliable change), 48% of TA group, 47% of CA group and 6% of control group participants were classified as having achieved reliable change at post-treatment on the PSWQ.

#### Treatment Satisfaction

Chi-squared tests and one-way ANOVAs failed to reveal any differences between treatment groups' ratings of satisfaction with the program with respect to: Overall satisfaction (p = .17); quality of the treatment lessons (p = .84); and quality of the support they received from the technician or clinician (p = .25). Overall, treatment group participants reported an acceptable level of satisfaction with the overall program, with 74/85 (87%) reporting being either *very satisfied* or *mostly satisfied*, and 11/85 (13%) *neutral/somewhat dissatisfied,* with 0% reporting *very dissatisfied*. Most responding participants (90%) rated the quality of the treatment modules as *excellent* or *good*, and 10% rated them as *satisfactory*; 83% rated the quality of contact with the clinician or technician as *excellent* or *good*, 15% rated it as *satisfactory,* and 2% as *unsatisfactory*.

When asked to provide a rating from 1 to 10, where 10 indicates a high level of agreement, the average participant rated the treatment as logical (9/10); they reported feeling confident that the treatment would be successful at teaching them techniques for managing their symptoms (8/10); and they reported a high level of confidence in recommending this treatment to a friend with GAD (9/10). No between treatment group differences were found on these items.

#### Time/Contact Events Per Participant

One-way ANOVAs revealed that each participant in the CA group received a greater mean (and SD) (33.2, 4.0) total number of contacts (telephone calls and emails) during the 8 week program than participants in the TA group (31.1, 3.1) (*F_1, 86_* = 7.94, p<0.01). However, no difference was found in the total mean (and SD) time spent by the technician (74.5 mins, 7.8) and clinician (80.8, 22.6) with each participant during the program (p = .08). These time estimates included monitoring individual progress, reading and responding to emails, discussing cases with the clinician, and attending weekly supervision sessions. Conducting this research added approximately 7 hours per week to the technician and the clinician's existing workload. The technician reported that four (10%) participants were discussed with the clinician who emailed these participants once only. No differences in pre-treatment symptom scores, demographic characteristics, or post-treatment symptom scores were observed between the TA group participants who were discussed with the clinician and the other participants.

### Follow-Up (3 Month) Outcomes

#### Primary Outcomes

A univariate ANCOVA controlling for pre-treatment scores (see Table 2), revealed that the CA group had significantly lower PSWQ scores at follow-up than the TA group (*F_1, 94_* = 5.01, p<0.03). A univariate ANCOVA, controlling for pre-treatment scores, failed to reveal differences between the CA and TA groups on the GAD-7 (p = 0.31). Paired samples t-tests for each intervention group revealed that the CA group made significant improvements between post-treatment and follow-up assessments on the PSWQ (t(40) = 2.72, p<0.01) but no change on the GAD-7 (p = 1.00), while the TA had no change on either PSWQ (p = 0.82) or GAD-7 (p = 0.58).

#### Secondary Outcomes

Univariate ANCOVAs conducted on the PHQ-9, K-10, and SDS follow-up scores, while controlling for pre-treatment scores, failed to reveal differences between the CA and TA groups on either the PHQ-9 (p = 0.92), K-10 (p = 0.49) or the SDS (p = 0.37). Paired samples t-tests for each intervention group failed to reveal any differences between post-treatment and follow-up for either the TA or CA groups on the secondary measures.

#### Effect Sizes

Pre to follow-up within-group effect sizes (Table 2) on the PSWQ were 1.42 and 0.97 for the clinician and technician-assisted groups, respectively, and 1.48 and 1.61 on the GAD-7, respectively. Large (>0.80) within-group effect sizes (ESs) were found for both treatment groups on the PHQ-9 and K-10, and for the TA group on the SDS.

#### Clinical Significance: Remission, Recovery, and Reliable Clinical Change

At follow-up (using the intention-to-treat and LOCF design), 10/50 (20%) of TA group and 9/47 (19%) of CA group participants continued to have a GAD-7 score above 9. Based on the criteria for recovery (a reduction of pre-treatment GAD-7 scores of at least 50%) at follow-up, 30/50 (60%) of TA group and 33/47 (70%) of CA group participants were classified as recovered. Based on the criteria for reliable clinical change (statistically reliable change), 42% of TA group and 59% of CA group were classified as having achieved reliable change at post-treatment on the PSWQ.

#### Differences Between 3-Month Completers and Non-Completers

Analyses were conducted to explore differences in pre-treatment, post-treatment, and changes scores of participants in the treatment groups who completed the follow-up questionnaires vs. participants who did not complete these measures. One-way ANOVAs revealed that the group who did not complete the 3-month follow-up questionnaires had significantly higher post-treatment PHQ-9 (*F_1, 45_* = 4.33, p<0.04), K-10 (*F_1, 45_* = 5.06, p<0.03), and SDS scores (*F_1, 45_* = 6.62, p<0.01), but no other differences were found (p range = 0.06–0.60).

## Discussion

This trial compared the efficacy and acceptability of technician- vs. clinician-assisted iCBT for GAD. At intake all participants met DSM-IV diagnosis of GAD, and the majority reported onset before the age of 30 years. In addition to access to the components of the Worry program, CA group participants had weekly email or telephone contact with the clinician and access to an online discussion forum. The clinician actively engaged in treatment with CA group participants. In addition to having access to the Worry program, TA group participants had weekly email or telephone contact with the technician, but did not have access to an online forum. The technician provided support and encouragement, did not provide clinical advice, but was instructed to refer clinical questions or concerns to the clinician.

The first hypothesis, that clinician-assisted treatment using the Worry program would be efficacious was supported. Large within-group effect sizes were obtained for the CA group on measures of GAD and importantly, satisfaction with treatment was high. This replicates the outcomes of an earlier preliminary study using the Worry program [27] and extends those results by confirming the stability or improvement of clinical gains 3 months post-program.

The second hypothesis, that participants in the TA group would show similar clinical improvements on measures of GAD, depression, and disability, to those in the CA group, was also supported. At post-treatment outcomes for both treatment groups were superior to the control group, satisfaction with treatment was high in both treatment groups, and there were no differences between the two treatment groups in clinical outcomes. At post-treatment more than 50% of participants in the treatment groups were classified as recovered compared to 10% of controls, while reliable clinical change was observed in almost 50% of participants in the treatment groups compared to 6% of controls. Consistent with the final hypothesis, at follow-up this pattern of results was maintained for participants in the TA group, while at follow-up the CA group obtained significantly lower scores on the PSWQ than at post-treatment, indicating that they made additional gains.

At post-treatment and follow-up ESs in the treatment groups on both GAD measures were greater than 1.0 indicating that the treatment effect was considerable. The magnitude of these ESs is comparable to improvements typically reported in meta-analyses of face-to-face CBT-based treatment of GAD [47], [48]. These results were obtained with a relatively low level of total contact time per participant but a large number of total contacts. It is estimated that 7 hours per week of clinician or technician time was required to conduct each group of more than 45 participants. However, during treatment CA group participants received approximately 33 contacts, prompts, and reminders compared to 31 in the TA group, a difference that was statistically, but unlikely to be clinically significant. More than half of these contacts were managed by the automated email system. These results indicate the importance of regular contact, even if the contact is automatic or of relatively short duration.

### Generalizability

These results replicate the findings of a recent RCT [27] reporting the preliminary results of the Worry program. The present results indicate the Worry program can reliably produce good clinical outcomes that are sustained for at least 3 months post-treatment, and that the procedure is acceptable to consumers with GAD. A total of 80 minutes of staff time was required per participant using the Worry program, which compares favorably to the 8 to 16 hours of clinician contact usually required in face-to-face treatment, indicating this approach is cost-effective. These results also support recent evidence indicating that Internet-based treatment programs may be effectively administered by a non-clinician [14], [25], [26], when supervised by a clinician. Importantly, if the technician in the present study was concerned about a participant in their group they were able to “step-up” participants to the clinician, but did so with only 10% of participants. This indicates that the majority found the intervention by the technician sufficient for their needs, and demonstrates a potential model for integrating clinician and technician support during iCBT programs.

### Limitations

The relatively small sample size is one limitation of this study. The low completion rates of questionnaires at follow-up is another limitation, and analyses revealed that non-completers had elevated post-treatment scores on the measures of depression, psychological distress, and disability relative to completers, indicating that the follow-up results should be interpreted with caution.

An important potential limitation is the use of a delayed treatment control group rather than an attention-control placebo. This choice was grounded in concerns about the impact of raised expectations of symptom resolution in anxious participants placed in a placebo, attention-control condition. These concerns were heightened by the geographical spread of participants, who were from all around Australia, and hence unable to be reached by the investigators should additional help have been required.

### Conclusions

This randomized controlled trial found no difference between a clinician- and technician-assisted Internet-based treatment program for GAD. Both conditions resulted in large effect sizes, clinically significant improvements, and high levels of acceptability, while a delayed treatment control group did not improve. These results were sustained at 3-month follow-up in the technician-assisted group, while the clinician-assisted group showed evidence of continued improvement. These findings are consistent with emerging evidence indicating that Internet-based treatment programs may be effectively administered by a non-clinician [14], [25], [26], when supervised by a clinician. Furthermore, this model of implementation requires considerably less time than face-to-face treatment, and appears highly acceptable to people with GAD. The question is not whether to accept such an innovative model of service delivery, but how to do so in an ethical, competent, safe, and cost-effective way, while maintaining excellent clinical standards.

## Supporting Information

Checklist S1 CONSORT Checklist
(0.19 MB DOC)

Protocol S1 Trial Protocol
(1.75 MB PDF)
