# Supplementary material for: Internet Treatment for Generalized Anxiety Disorder: A Randomized Controlled Trial Comparing Clinician vs. Technician Assistance
Source: PLoS One. 2010 Jun 3;5(6):e10942. doi: 10.1371/journal.pone.0010942 (PMC2880592; doi:10.1371/journal.pone.0010942)
Supplement: Protocol S1 — Trial Protocol (1.75 MB PDF) [file pone.0010942.s002.pdf]

Online Forms
National Ethics Application Form

Within which organisations will the research sites be located: *(tick all that apply)*

- [x] New South Wales
- [ ] Queensland

Name/ID of HREC reviewing the research project:
St Vincent's Hospital Human Research Ethics Committee (EC00140)

HREC Application Reference Number:

Submission date:
22/06/2009

1. TITLE AND SUMMARY OF PROJECT

1. Title

What is the formal title of this research proposal?
Distance treatment III: Randomised controlled trial in Generalized Anxiety Disorder

What is the short title / acronym of this research proposal (if applicable)?
Distance treatment III: RCT of the Worry Program

2. Description of the project in plain language

Give a concise and simple description (not more than 400 words), in plain language, of the aims of this project, the proposal research design and the methods to be used to achieve those aims.

Many people with generalized anxiety disorder (GAD) do not access treatment. We have developed an Internet based treatment program for GAD, which is currently being piloted in an RCT (treatment vs waitlist control). Preliminary results indicate that this treatment program is effective.

This proposed phase 2b study will recruit 120 people with GAD and randomly allocate them to one of three groups: (1) Clinician guided internet–based treatment group, (2) Self–guided internet–treatment group, (3) Wait–list control group (who will receive treatment after the treatment groups have completed the program). We will measure changes in symptom level in all three groups from the beginning of the 10 week program course of treatment and one week after the conclusion of treatment. The two treatment groups will also complete the follow up questionnaires at 3 months post treatment. We will also measure adherence to the lessons, homework and forum, satisfaction with the clinician's input and satisfaction with the mode of treatment generally.

This proposed project employs the same research design as that previously approved by St Vincent's Hospital Human Research Ethics Committee ((EC00140)(AB/3097/1)

The questionnaires being administered in this study pre and post treatment are:

GAD–7 (Short measure of Generalised Anxiety Disorder. Spitzer R, Kroenke, K, Williams J, Löwe, B(2006)).
PSWQ (Penn State Worry Questionnaire (Meyer et al, 1990)).
PHQ–9: Patient Health Questionnaire – Nine Item (Kronke K, Spitzer R, Williams J, 1001).
K–10 and WHODAS–II: Kessler–10 and World Health Organisation Disability Assessment Schedule (Kessler R, Andrews G, Colpe L et al (2002)).
SIAS: Social Interaction Anxiety Scale (Mattick R, & Clarke J (1998))
SDS: Sheehan Disability Scale (Sheehan, 1983).
CEQ: Credibility/Expectancy Questionnaire (Devilly G, Borkovec T (2000)).

2. RESEARCHERS / INVESTIGATORS

1. Chief researcher(s)/investigator(s)

Chief researcher

	Title:	Forename/Initials:	Surname:
	Dr	Nickolai	Titov

Mailing Address: 299 Forbes Street

Suburb/Town: Darlinghurst
State: NSW
Postcode: 2010
Country: Australia
Organisation: UNSW at St Vincent's Hospital, Sydney
Department*: School of Psychiatry
Position: Senior Lecturer
E-mail: nickt@unsw.edu.au
Phone (BH): 02 83821732
Phone (AH)*: 02 83821732
Mobile*:
Pager*:
Fax: 02 83821721

Is this person the contact person for this application?
(●) Yes ( ) No

Summary of qualifications and relevant expertise.
PhD, registered clinical psychologist with special interest in internet treatment. Developed the model for distance internet treatment using clinician assisted CBT.

Please declare any general competing interests.
N/A

Name the site(s) for which this chief researcher / investigator is responsible.
St Vincent's Hospital VirtualClinic, Sydney

Describe the role of the chief researcher / investigator in this project.
Will have responsibility for the running of this project.

Is the chief researcher / investigator a student? ( ) Yes (●) No

2. Principal researcher(s) / investigator(s)

Principal researcher / investigator 1

Title:	Forename/Initials:	Surname:
Professor	John Gavin	Andrews

Mailing Address: 299 Forbes Street

Suburb/Town: Darlinghurst
State: NSW
Postcode: 2010
Country: Australia
Organisation: UNSW at St Vincent's Hospital, Sydney
Department*: Clinical Research Unit for Anxiety and Depression
Position: Professor
E-mail: gavina@unsw.edu.au

Phone (BH): 02 83821726
Phone (AH)*:
Mobile*:
Pager*:
Fax: 02 83821721

Is this person the contact person for this application?
◯ Yes ◉ No

Summary of qualifications and relevant expertise
MD FRCPsych FRANZCP. Authored numerous books and journal articles on anxiety and depression treatment.

Please declare any general competing interests
N/A

Name the site(s) for which this principal researcher / investigator is responsible.
St Vincent's Hospital, Sydney

Describe the role of the principal researcher / investigator in this project.
Advise the associate investigators.

Is the principal researcher a student? ◯ Yes ◉ No

3. Associate Researcher(s) / investigator(s)

How many known associate researchers are there? (You will be asked to give contact details for these associate researchers / investigators) 1

Do you intend to employ other associate researchers / investigators? ◯ Yes ◉ No

Associate Researcher / Investigator 1

Title:	Forename/Initials:	Surname:
Dr	Emma	Robinson

Mailing Address: 299 Forbes Street

Suburb/Town: Darlinghurst
State: NSW
Postcode: 2010
Country: Australia
Organisation: St Vincent's Hospital, Sydney
Department*: Clinical Research Unit for Anxiety and Depression
Position: Clinical Psychologist
E–mail: erobinson@stvincents.com.au
Phone (BH): 02 83821729
Phone (AH)*:
Mobile*:
Pager*:
Fax: 02 83821721

Is this person the contact person for this application?
◯ Yes ◉ No

Summary of qualifications and relevant expertise
PsyD. Clinical psychologist with experience in cognitive behaviour therapy for anxiety and depression. Experience in online treatment of people with GAD.

Please declare any general competing interests
N/A

Description of the role of the associate researcher / investigator in this

project.
Conduct the treatment of people with GAD.

Name the site at which the associate researcher / investigator has responsibility.
St Vincent's Hospital, Sydney.

Is this associate researcher / investigator a student? ○ Yes ◉ No

5. Other personnel relevant to the research project

5a. How many known other people will play a specified role in the conduct of this research project?

1

5b. Describe the role, and expertise where relevant (e.g. counsellor), of these other personnel.

Karen Solley – Research Assistant, B Sc (Hons in Psychology)

5c. Is it intended that other people, not yet known, will play a specified role in the conduct of this research project?

○ Yes ◉ No

6. Certification of researchers / investigators

6a. Are there any relevant certification, accreditation or credentialing requirements relevant to the conduct of this research?

○ Yes ◉ No

7. Training of researchers

7a. Do the researchers / investigators or others involved in any aspect of this research project require any additional training in order to undertake this research?

◉ Yes ○ No

What is this training?
Dr Robinson will be provided with an instructional manual pertinent to operating the online GAD program. Protocol training will also be undertaken after ethical approval is granted to ensure that all roles and responsibilities of those involved are clear and understood as per this application. This will occur prior to the commencement of the trial.

How and by whom will the training be provided?
Dr Titov will provide this training once ethical approval has been received.

How will the outcome of the training be evaluated?
Competency tests will be administered by Dr Titov evaluating ability to use the software required.

3. RESOURCES

Project Funding / Support

1. Indicate how the project will be funded?

Type of funding.

[Please note that all fields in any selected funding detail column (with the exception of the code) will need to be completed.]

Funding	Confirmed or Sought?
External Competitive Grant	(●) Confirmed ( ) Sought ( ) Not Sought *Amount of funding* $38 362
Internal Competitive Grant	( ) Confirmed ( ) Sought ( ) Not Sought *Amount of funding*
Sponsor	( ) Confirmed ( ) Sought ( ) Not Sought *Amount of funding*
By Researchers Department or Organisation	( ) Confirmed ( ) Sought (●) Not Sought

1a. External Competitive Grant

Name of Grant / Sponsor	Australian Rotary Health Research Fund 2008
Code (optional)	
Detail in kind support	This funding was matched with funding from our Department to cover costs of a research assistant to with participant recruitment.
Indicate the extent to which the scope of the grant and the scope of this HREC application are aligned:	This grant is to provide partial costs of a research assistant for this project.

1b. Internal Competitive Grant

Name of Grant / Sponsor

Code (optional)

Detail in kind support

Indicate the extent to which the scope of the grant and the scope of this HREC application are aligned:

1c. Sponsor

Name of Grant / Sponsor

Code (optional)

Detail in kind support

Indicate the extent to which the scope of the grant and the scope of this HREC application are aligned:

2. How will you manage a funding shortfall. (if any)

CRUfAD has trust funds that would cover any shortfall.

3. Will the project be supported in other ways eg. in-kind support/equipment by an external party eg. sponsor?

( ) Yes (●) No

4. Is this a study where capitation payments are to be made, and will participants be made aware of these payments to clinicians or researchers / investigators?

N/A

Duality of Interest

5. Describe any commercialisation or intellectual property implications of the funding/support arrangement.

The CaCCBT and the CCBT programs are the property of St Vincent's Hospital

6. Does the funding/support provider(s) have a financial interest in the outcome of the research?

○ Yes ◉ No

7. Does any member of the research team have any affiliation with the provider(s) of funding/support, or a financial interest in the outcome of the research?

◉ Yes ○ No

Describe affiliation(s) and/or interest(s):
The Hospital owns it and employs the staff
Do you consider the relationship between the research team and the funding/support provider constitutes:

- ☐ a potential conflict of interest
- ☐ a potential duality of interest
- ☑ no ethical issue

Provide an explanation:
The hospital treats people for anxiety and this is a program to do that.

8. Does any other individual or organisation have an interest in the outcome of this research?

◉ Yes ○ No

Indicate the interested party and describe the interest:
All three researchers are affiliated with the Hospital.

9. Are there any restrictions on the publication of results from this research?

○ Yes ◉ No

4. PRIOR REVIEWS

Ethical Review

Some HRECs may require researchers to provide information additional to that contained in a NEAF proposal. For this reason, it is prudent to check whether the HRECs to whom you propose to submit this proposal require additional information.

Duration and location

1. In how many Australian sites, or site types, will the research be conducted?

1

2. In how many overseas sites, or site types, will the research be conducted?

0

3. Provide the following information for each site or site type (Australian and overseas, if applicable) at which the research is to be conducted

1

Site / Site Type Name:	St Vincent's Hospital
Site / Site Type Location:	390 Victoria St<br>Darlinghurst, 2010<br>NSW

4. Provide the start and finish dates for the whole of the study including data analysis

Anticipated start date: 01/09/2009 *(dd/mm/yyyy)*
Anticipated finish date: 01/04/2010 *(dd/mm/yyyy)*

5. Are there any time–critical aspects of the research project of which an HREC should be aware?

○ Yes ◉ No

6. To how many Australian HRECs (representing site organisations or the researcher's / investigator's organisation) is it intended that this research proposal be submitted?

1

A list of NHMRC registered Human Research Ethics Committees (HRECs), along with their institutional affiliations and contact details is available on the NHMRC website at the following web address: http://www.nhmrc.gov.au/health_ethics/hrecs/overview.htm#d.

7. HRECs

HREC 1

Name of HREC:
St Vincent's Hospital Human Research Ethics Committee (EC00140)

Provide the start and finish dates for the research for which this HREC is providing ethical review:
Anticipated start date or date range: 01/09/2009 *(dd/mm/yyyy)*
Anticipated finish date or date range: 01/04/2010 *(dd/mm/yyyy)*

For how many sites at which the research is to be conducted will this HREC provide ethical review?
1

Site 1

Name of Site: St Vincent's Hospital

Principal Researcher 1

Principal Researcher Name:
Dr Nickolai Titov

Associate Researcher 1

Associate Researcher Name:
Professor John Gavin Andrews

Associate Researcher 2

Associate Researcher Name:
Dr Emma Robinson

8. Have you previously submitted an application, whether in NEAF of otherwise, for ethical review of this research project to any other HRECs?

○ Yes ◉ No

9. HRECs

Research conducted overseas

Peer review

11. Has the research proposal, including design, methodology and evaluation undergone, or will it undergo, a peer review process?

◉ Yes ○ No

Provide details of the review and the outcome. A copy of the letter / notification, where available, should be attached to this application.
It was approved at the weekly research meeting of the Clinical Research Unit for Anxiety and Depression.

5. PROJECT

1. Type of Research

Tick as many of the following 'types of research' as apply to this project. Your answers will assist HRECs in considering your proposal. A tick in some of these boxes will generate additional questions relevant to your proposal (mainly because the National Statement requires additional ethical matters to be considered), which will appear in Section 4 of NEAF.

The project involves:

- [ ] Research using qualitative methods
- [ ] Research using quantitative methods, population level data or databanks, e.g survey research, epidemiological research
- [x] Clinical research
- [ ] Research involving the collection and / or use of human samples
- [ ] Genetic testing/research
- [ ] A cellular therapy
- [ ] Research on workplace practices or possibly impacting on workplace relationships
- [ ] Research conducted overseas involving participants
- [ ] Research involving ionising radiation
- [ ] Research involving gametes or use or creation of embryos
- [ ] None of the above

Does the research involve limited disclosure to participants?
( ) Yes (•) No

Are the applicants asking the HREC / review body to waive the requirement of consent?
( ) Yes (•) No

Research plan

2. Describe the theoretical, empirical and/or conceptual basis, and background evidence, for the research proposal, eg. previous studies, anecdotal evidence, review of literature, prior observation, laboratory or animal studies.

Many people with anxiety disorders do not access treatment (Andrews et al, 2004). The reason for the shortfall is well known as being a mixture of societal, attitudinal and diagnostic variables (Andrews et al, 2001). Making treatment more freely available in areas where expert treatment is not available, or to people who are unable to take time off work to access treatment is one logical step. This was the aim of a series of studies focusing on Social Phobia, Depression and Panic Disorder (previously approved by St Vincent's Hospital Human Research Ethics Committee (EC00140)(AB/3097/1)). The current study aims to contribute to this series by addressing generalised anxiety disorder (GAD).

Generalised Anxiety Disorder responds to cognitive behaviour therapy (CBT) and to medication with SSRIs (Nathan & Gorman, 2007)). CBT for this disorder can be delivered over the internet, but one difficulty with computerised CBT (CCBT) is that adherence is usually extremely poor in the absence of clinician input (Titov, 2007). We recently completed a randomised controlled trial of clinician assisted computerised CBT (CaCCBT) for the distance treatment of people with social phobia (Titov, et al, 2008). Adherence was 80%. Patients in the intervention groups were as severe as patients seen in the specialised anxiety disorders clinic at St Vincent's Hospital and yet made the same level of improvement as did people receiving face to face treatment (ES respectively 0.8 clinic, 1.2 CaCCBT,)however the latter group only required one quarter as much clinician time. This finding has been replicated (Titov, Andrews and Schwencke, 2008).

The aim of this study is to determine the effectiveness of clinican guided internet–based treatment compared to self–guided internet treatment compared against wait–list control for generalised anxiety disorder.

3. State the aims of the research and the research question and/or hypotheses, where appropriate.

To explore the outcomes of the clinician assisted CBT programs for people with Generalised Anxiety Disorder compared to the status of people who receive CCBT or who remain on the wait list using a RCT design.

To further determine the feasibility of these treatments in terms of acceptability to patients and practicality for clinicians.

4. Has this project been undertaken previously?

○ Yes ◉ No

Benefits/Risks

In answering the following questions (Q 5 – 11) please ensure that you address all issues relevant to the type of participants that will be involved in your research project. Refer for guidance to relevant chapters of the National Statement.

5. Does the research involve a practice or intervention which is an alternative to a standard practice or intervention?

◉ Yes ○ No

Explain how the practice or intervention differs from standard practice or intervention:
The research involves treatment over the internet. While such offers of treatment are common, such treatment supported by a skilled clinician attending to the needs of the individual patient is very uncommon, even if desirable. There will be telephone contact with the participants. Diagnostic questionnaires will be administered over the phone by Dr Emma Robinson and Karen Solley (Research Assistant).

7. What expected benefits (if any) will this research have for the wider community?

If the trial is successful the Hospital would consider offering this type of treatment as a routine to the benefit of the wider community.

8. What expected benefits (if any) will this research have for participants?

We would expect that the average participant in the CaCCBT group would improve by 1 SD+ on standardised measures of Generalised Anxiety Disorder. In practical terms this means that a clinically significant reduction in symptoms of generalised anxiety disorder will have occured, similar to those found in face to face treatment.

9. Are there any risks to participants as a result of participation in this research project?

◉ Yes ○ No

10. Explain how the likely benefit of the research justifies the risks of harm or discomfort to participants.

Web based CBT is beneficial when people adhere to the program (Titov, 2007). This research will foster adherence by good clinical guidance. We therefore expect people to benefit from the program which justifies any discomfort experienced during the course.

11. Are there any other risks involved in this research? eg. to the research team, the organisation, others

○ Yes ◉ No

12. Is it anticipated that the research will lead to commercial benefit for the investigator(s) and or the research sponsor(s)?

○ Yes ◉ No

16. Is there a risk that the dissemination of results could cause harm of any kind to individual participants – whether their physical, psychological, spiritual, emotional, social or financial well-being, or to their employability or professional relationships – or to their communities?

○ Yes ◉ No

Monitoring

17. What mechanisms do the researchers / investigators intend to implement to monitor the conduct and progress of the research project?

Patient safety will be monitored on a daily basis. Regular team meetings (at least 3 times a week) will be conducted to monitor any difficulties patients may be having and ways of best dealing with these difficulties. Consent will also be monitored and any patients who wish to withdraw will be able to do this and be provided with other options at any time. Adverse events will be monitored by the investigators and reported to the ethics committee.

Patients' progress is monitored by their feedback on homework activities and postings on forums. Patients who do not participate for more than two weeks will be contacted, first by email and then by phone. If they remain out of contact, and there is cause for concern, their general practitioner will be contacted. These patient issues will be discussed at the regular team meetings.

18. Please detail your DSMB and its nominee for this trial.

We do not have a DSMB for this trial as it is not a drug trial.

6. PARTICIPANTS

1. Research participants

The National Statement identifies the need to pay additional attention to ethical issues associated with research involving certain specific populations.

This question aims to assist you and the HREC to identify and address ethical issues that are likely to arise in your research, if its design will include one or more of these populations. Further, the National Statement recognizes the cultural diversity of Australia's population and the importance of respect for that diversity in the recruitment and involvement of participants. Your answer to this question will guide you to additional questions (if any) relevant to the participants in your study.

Tick as many of the following 'types of research participants' who will be included because of the project design, or their inclusion is possible, given the diversity of Australia's population. If none apply, please indicate this below.

If you select column (a) or (b), column (c) will not apply.

**The participants who may be involved in this research are:** *If you select column (a) or (b), column (c) will not apply.*	**a) Primary intent of research**	**b) Probable coincidental recruitment**	**c) Design specifically excludes**
People whose primary language is other than English (LOTE)	☐	☑	☐
Women who are pregnant and the human foetus	☐	☐	☐
Children and/or young people (ie. <18 years)	☐	☐	☑
People in existing dependent or unequal relationships	☐	☐	☐
People highly dependent on medical care	☐	☐	☐
People with a cognitive impairment, an intellectual disability or a mental illness	☐	☐	☑
Aboriginal and/or Torres Strait Islander peoples	☐	☐	☐
People who may be involved in illegal activity	☐	☐	☐
None apply	☐		

You have indicated that it is probable that
– People whose primary language is other than English (LOTE)
may be coincidentally recruited into this project. The National Statement identifies specific ethical considerations for these groups(s).

Please explain how you will address these considerations in your proposed research.
That their primary language is not English is not of concern. They will however need to be able to read and write English at a School Certificate level.

The research will be conducted in English. If the individual can understant the recruitment process they are likely to be able to adequately participate.

Participant description

2. How many participant groups are involved in this research project?

3

3. What is the expected total number of participants in this project at all sites?

120

4. Groups

Group 1

Group name for participants in this group:	Clinician Assisted Cognitive Behavioural Therapy (CaCCBT)
Expected number of participants in this group:	40
Age range:	18+

Other relevant characteristics of this participant group:
– All will meet criteria for Generalised Anxiety Disorder.
– All will have been randomised into this Group
Why are these characteristics relevant to the aims of the project?
– This is a study of the efficacy of web–based treatment for Generalised Anxiety Disorder
– This group is being compared to 2 other treatment groups – Self Guided CBT and Waitlist Control.

Group 2

Group name for participants in this group:	Self–Guided Cognitive Behavioural Therapy (CCBT)
Expected number of participants in this group:	40
Age range:	18+

Other relevant characteristics of this participant group:
– All will meet criteria for Generalised Anxiety Disorder.
– All will have been randomised into this Group
Why are these characteristics relevant to the aims of the project?
– This is a study of the efficacy of treatment for Generalised Anxiety Disorder
– This group is being compared to 2 other treatment groups – CaCCBT and Waitlist control.

Group 3

Group name for participants in this group:	Waitlist Control
Expected number of participants in this group:	40
Age range:	18+

Other relevant characteristics of this participant group:
– All will meet criteria for Generalised Anxiety Disorder.
– All will have been randomised into this Group
Why are these characteristics relevant to the aims of the project?
– This is a study test of the efficacy of treatment for Generalised Anxiety Disorder
– This group is being compared to 2 other treatment groups – Clinician Assisted CBT and CCBT.

Your response to question 1 at Section 6 – "Research Participants" indicates that the following participant groups are excluded from your research. If this is not correct please return to question 1 at Section 6 to amend your answer.

- Children and/or young people (ie. <18 years)

- People with an intellectual or mental impairment

5. Have any particular potential participants or groups of participants been excluded from this research? In answering this question you need to consider if it would be unjust to exclude these potential participants.

We need more data on the efficacy of these treatments in young people before inviting their participation. Future research will do this.

We need more data on the efficacy of these treatments in people with intellectual impairment before inviting their participation.

Participant experience

6. Provide a concise detailed description, in not more than 200 words, in terms which are easily understood by the lay reader of what the participation will involve.

They will identify themselves as suffering from Generalised Anxiety Disorder, and we will confirm this by questionnaire and by interview. The program will provide for the CaCCBT group, through illustrated story lines, homework exercises, postings on the forum and advice from the clinician, instruction about recovering from their disorder. They will then redo the questionnaires and receive supportive advice about their progress and additional needs for treatment, if any. The CCBT group will not receive the clinican support but will have access to lessons, the wait list group will receive nothing during that period. All three groups will do the exit questionnaires. Patients in the CCBT and waitlist groups will be offered CaCCBT at the end of the study if it proves to be useful.

Relationship of researchers / investigators to participants

7. Specify the nature of any existing relationship or one likely to rise during the research, between the potential participants and any member of the research team or an organisation involved in the research.

The clinician will have, as a result of the patients' application for treatment, a clinician–patient relationship. The patients will become patients of St Vincent's Hospital.

9. Describe what steps, if any, will be taken to ensure that the relationship does not impair participants' free and voluntary consent and participation in the project.

Their consent remains free and voluntary and they can withdraw at any time.

10. Describe what steps, if any, will be taken to ensure that decisions about participation in the research do not impair any existing or foreseeable future relationship between participants and researcher / investigator or organisations.

This is distance treatment and few, if any, will have other needs for services from the Anxiety Disorders Clinic. They do however retain the right to apply for face to face treatment in the normal way.

11. Will the research impact upon, or change, an existing relationship between participants and researcher / investigator or organisations?

○ Yes ◉ No

Recruitment

13. What processes will be used to identify potential participants?

There are currently 200 people on the waitlist at our website (www.virtualclinic.org.au). Patients self–identify from looking at the website virtualclinic.org.au and they then voluntarily apply for the program. Some patients may also hear about the program through some media coverage which explains our projects. We do not intend to place advertisements.

14. Is it proposed to 'screen' or assess the suitability of the potential participants for the study?

◉ Yes ○ No

How will this be done?

Initially this is in terms of self identified main problem. This is then confirmed by questionnaire measures and a telephone interview in which a brief psychiatric interview is conducted.

15. Describe how initial contact will be made with potential participants.

Interested people will log on to the web site www.climateclinic.tv / www.virtualclinic.org.au, read about the study and decide to apply. Some 500 people responded to the Phase 2 trial for Social Phobia in 2008 following some general media coverage. We still receive some 5 to 10 enquiries per week. We would anticipate a similar rate of contact.

16. Do you intend to include both males and females in this study?

(•) Yes ( ) No

What is the expected ratio of males to females that will be recruited into this study and does this ratio accurately reflect the distribution of the disease, issue or condition within the general community?
We would anticipate more females than males to be recruited into the study. This reflects the higher proportion of females represented in populations suffering from GAD, (Hunt et al., 2002, Carter et al., 2001). This was also our experience with recruiting for the shyness program.

17. Is an advertisement, e–mail, website, letter or telephone call proposed as the form of initial contact with potential participants?

( ) Yes (•) No

18. If it became known that a person was recruited to, participated in, or was excluded from the research, would that knowledge expose the person to any disadvantage or risk?

( ) Yes (•) No

Consent process

19. Will consent for participation in this research be sought from all participants?

(•) Yes ( ) No

Will there be participants who have capacity to give consent for themselves?
(•) Yes ( ) No

What mechanisms/assessments/tools are to be used, if any, to determine each of these participant's capacity to decide whether or not to participate?
The study will be described on the intake website. People will elect to continue or not. Only people whose responses to the questions are internally consistent will proceed to the second stage of diagnostic questionnaires and interview.

The diagnostic interview provides a further safeguard against wrongful inclusion.

Are any of the participants children or young people?
( ) Yes (•) No

Will there be participants who do not have capacity to give consent for themselves?
( ) Yes (•) No
The following questions relate to participants who are able to provide consent and also to participants for whom consent may be provided by a person with legal authority to do so. When answering these questions you need to describe any differences in the processes followed, or the documentation used, for different groups of participants in your proposal, e.g. processes and documentation for users of facilities/services will differ from those for providers of those facilities/services. Where your proposal involves participants with an intellectual or mental impairment, or people in dependent relationships, additional questions about their consent appear at section 7 questions 19–20 and questions 15–18 respectively.

Describe the consent process, ie how participants or those deciding for them will be informed about, and choose whether or not to participate in, the project. The study will be described on the intake website. People will elect to continue or not, screening as to suitability will be automated. Only people whose responses to the questions match criteria and are internally consistent with the diagnostic criteria will proceed to the second stage of diagnostic questionnaires and interview. Participants registering for final consideration will read the information sheet and sign a

consent form and mail that to the investigators. This will trigger the phone interview in which the diagnosis will be confirmed, questions about the study answered, and an offer of treatment made.
If a participant or person on behalf of a participant chooses not to participate, are there specific consequences of which they should be made aware, prior to making this decision? No.
Might individual participants be identifiable by other members of their group, and if so could this identification could expose them to risks? No
If a participant or person on behalf of a participant chooses to withdraw from the research, are there specific consequences of which they should be made aware, prior to giving consent? No
Specify the nature and value of any proposed incentive/payment (eg. movie tickets, food vouchers) or reimbursement (eg travel expenses) to participants. None
Explain why this offer will not impair the voluntary nature of the consent, whether by participants' or persons deciding for their behalf. N/A

Do you propose to obtain consent from individual participants for your use of their stored data/samples for this research project?
(•) Yes ( ) No

7. Participants Specific

8. CONFIDENTIALITY/PRIVACY

Answers to the questions in section 8.1 will establish whether an HREC will need to apply guidelines under federal or State/territory privacy legislation in reviewing your application. Answers to questions in the remaining parts of section 8 will show how confidentiality of participants is to be protected in your research.

1. Do privacy guidelines need to be applied in the ethical review of this proposal?

Indicate whether the source of the information about participants which will be used in this research project will involve:

- [x] collection directly from the participant
- [ ] collection from another person about the participant
- [ ] use or disclosure of information by an agency, authority or organization other than your organisation
- [ ] use of information which you or your organisation collected previously for a purpose other than this research project

Information which will be collected for this research project directly from the participant

Describe the information that will be collected directly from participants. Be specific where appropriate. Name and address/phone number, age, gender, and demographic details.

Self identified principal disorder, confirmed by telephone diagnostic interview with the Generalised Anxiety Disorder section of the Mini International Neuropsychiatric Interview.

Information about psychotic symptoms and substance abuse for purposes of exclusion.

Scores on the relevant generalised anxiety symptom scales and on the general measures of stress and of disability.

The information collected by the research team about participants will be in the following form(s). Tick more than one box if applicable.
- [x] individually identifiable
- [ ] re-identifiable
- [ ] non-identifiable

Give reasons why it is necessary to collect information in individually identifiable or re-identifiable form

They will be patients of the Hospital. This information is collected as part of their distance treatment and the details will be recorded in their hospital medical record. Hospital records are usually identified.
Furthermore, the clinician will need to collect identified information from the patient in order to monitor their progress as part of good clinical practice.

Using information from participants

2. Describe how information collected about participants will be used in this project.

Scores will be used to determine suitability for treatment and then to calculate their improvement with treatment. This knowledge will enable the clinician to debrief each patient appropriately with advice as to a prudent next step in treatment if required when the trial is over.

3. Will any of the information be used by the research team be in identified or re-identifiable (coded) form?

(●) Yes ( ) No

Indicate whichever of the following applies to this project:

- [x] Information collected for, used in, or generated by, this project will not be used for any other purpose.
- [ ] Information collected for, used in, or generated by, this project will/may be used for another purpose by the researcher for which ethical approval will be sought.
- [ ] Information collected for, used in, or generated by, this project is intended to be used for establishing a database/data collection/register for future use by the researcher for which ethical approval will be sought.
- [ ] Information collected for, used in, or generated by, this project will/may be made available to a third party for a subsequent use for which ethical approval will be sought.

4. List ALL research personnel and others who, for the purposes of this research, will have authority to use or have access to the information and describe the nature of the use or access. Examples of others are: student supervisors, research monitors, pharmaceutical company monitors.

Only the responsible clinician will see identified data. De–identified data will be seen by all three investigators and the research assistant in the analysis and write up phases. Some of our previous RCT patients gave permission for some of their deidentified postings on the forum to be displayed to help others. We presume that this will occur in this trial.

Storage of information about participants during and after completion of the project

5. In what formats will the information be stored during the research project? (eg. paper copy, computer file on floppy disk or CD, audio tape, videotape, film)

Identified data will be stored securely on a password protected computer, de–identified data will be stored as a computer file with back up.
We will also create a paper medical record that will contain name, address, phone number and email address plus summary scores on assessment scales before and after treatment.

6. Specify the measures to be taken to ensure the security of information from misuse, loss, or unauthorised access while stored during the research project? (eg. will identifiers be removed and at what stage? Will the information be physically stored in a locked cabinet?)

Identified and de–identified data will be password protected from unauthorised access. Identifiers will be removed when the scores are transferred from the individual patient records to the data base to form the de–identified data. Identified data (medical records) will become the property of the Hospital.

9. The information which will be stored at the completion of this project is of the following type(s). Tick more than one box if applicable.

- [x] individually identifiable
- [ ] re–identifiable
- [x] non–identifiable

Give reasons why it is necessary to store information in individually identifiable or re–identifiable form.
The records form part of the patients medical record, hospital records are identifiable.

10. For how long will the information be stored after the completion of the project and why has this period been chosen?

For 15 years consistent with guidelines of storage of clinical materials.

11. What arrangements are in place with regard to the storage of the information collected for, used in, or generated by this project in the event that the principal researcher / investigator ceases to be engaged at the current organisation?

His successor will be given the responsibility to fufil the ethical obligations as outlined in this application form.

Ownership of the information collected during the research project and resulting from the research project

13. Who is understood to own the information resulting from the research, eg. the final report or published form of the results?

The three investigators

14. Does the owner of the information or any other party have any right to impose limitations or conditions on the publication of the results of this project?

( ) Yes (•) No

Disposal of the information

15. Will the information collected for, used in, or generated by this project be disposed of at some stage?

(•) Yes ( ) No

At what stage will the information be disposed?
15years
How will information, in all forms, be disposed?
Paper records will be disposed of via medical record clearance; the computer database will be erased after 15 years.

Reporting individual results to participants and others

16. Is it intended that results of the research that relate to a specific participant be reported to that participant?

(●) Yes ( ) No

Specify in what form the results will be reported to participants:
The treating clinician will conduct an email interchange with that participant.
How will the results be communicated to participants? eg telephone call, individual letter, copy of publication, consultation with a medical practitioner or other
Email.
Who will be responsible for communicating the project results to participants?
The treating clinician

17. Is the research likely to produce information of personal significance to individual participants?

(●) Yes ( ) No

18. Will individual participant's results be recorded with their personal records?

(●) Yes ( ) No

19. Is it intended that results that relate to a specific participant be reported to anyone other than that participant?

(●) Yes ( ) No

To whom will the results be reported other than the participant?
Participants will be required to nominate a GP to whom we can report. We will do this should there be reasons for concern, i.e. sustained high depression scores (PHQ-9 >20)
Explain why the results will be reported to a person other than the participant?
We will have permission to communicate with their own doctor.
Will the participant be told that their results will be reported to another person?
(●) Yes ( ) No

20. Is the research likely to reveal a significant risk to the health or well being of persons other than the participant, eg family members, colleagues

( ) Yes (●) No

21. Is there a risk that the dissemination of results could cause harm of any kind to individual participants – whether their physical, psychological, spiritual, emotional, social or financial well-being, or to their employability or professional relationships – or to their communities?

( ) Yes (●) No

22. How is it intended to disseminate the results of the research? eg report, publication, thesis

In journal articles and at conferences

23. Will the confidentiality of participants and their data be protected in the dissemination of research results?

(●) Yes ( ) No

Explain how confidentiality of participants and their data will be protected in the dissemination of research results:
Only deidentified data will be available to the researchers when they analyse the data, only grouped data will be reported.

9. PROJECT SPECIFIC

Your responses to question 5.1 "Type of Research" and question 6.1 "Research participants" indicate that the HREC will require additional information which is specific to your research project. The following table indicates the question sets relating to the project that you will need to complete. If this is not correct please return to question 5.1 and 6.1 at to amend your answer.

- 9.1. Type of research/trial
- 9.2. Clinical research

9.1 Type of research/trial

1. The study involves:

- ☐ The administration of a drug / medicine (includes a complementary / alternative medicine)
- ☐ The use of a medical device
- ☐ The administration of human somatic cell gene therapy
- ☐ The use of a xenotransplant
- ☐ The use of stem cells (adult or embryonic) as therapy
- ☑ Other

*Describe the type of study to be conducted:*An RCT of Clinician Assisted Computerised CBT (CaCCBT) vs Computerised CBT (CCBT) vs Waitlist Control.

NB: This is not a drug trial it is a psychological educational program.

2. The project will be conducted as follows:

Under the Clinical Trial Notification Scheme (CTN) ○ Yes ◉ No

Under the Clinical Trial Exemption Scheme (CTX) ○ Yes ◉ No

You have indicated that you are conducting a clinical trial under <u>neither</u> the CTN or CTX scheme. Please ensure that this is correc referring back to your answer at Page 16, Section 5, Question 1 'Type of Research' If you are conducting a trial in a clinical setting which will not take place under CTN or CTX, please ensure that enough detail has been provided about the research to allow a HI to adequately review it. This may require you to review your answers in Page 16, Section 5, Question 1 Type of Research and/or I 20, Section 6, Question 1 Research participants

3. Provide the following details for the clinical trial protocol:

Protocol name:	Distance treatment III: RCT of the Worry Program.
Protocol version number:	1.1
Protocol version date:	17/06/2009 *(dd/mm/yyyy)*

*If you intend to/have registered this trial in a publicly accessible register, please provide the details of it here*It will be registered on ANZCTR.

NB: This is not a drug trail, it is a psychological educational program.

4. Provide the following details for the investigator's brochure/product information (as relevant):

Title of Investigator's Brochure:	n/a
Investigator's brochure version number:	n/a
Investigator's brochure version date:	*(dd/mm/yyyy)*

9.2 Clinical research

1. The study examines:

☐ The administration of a drug / medicine (includes a complementary / alternative medicine)

☐ The use of a medical device

☑ Other

*Describe briefly the type of study to be conducted:*An RCT of Clinician Assisted Computerised CBT vs Computerised CBT vs Waitlist Control

2. Provide the following details for the study protocol:

Protocol title:	RCT of the Worry Program
Protocol version number:	1.1
Protocol version date:	16/06/2009 *(dd/mm/yyyy)*

3. Provide a statement addressing the following as may be applicable to the project.

a) Method of randomisation
b) Whether the hypothesis offers a realistic possibility that the intervention is at least as effective as standard treatment
c) The justification for the use of placebo or non–treatment control group, including alternative effective treatments and any risk of harm in the absence of treatment.
d) How variations in response will be treated
e) Endpoints
f) Details of contingencies and management of these
g) Explain the arrangements in place to ensure there is adequate compensation for participants.

a) Using www.random.org

b) The hypothesis does offer a realistic possibility that the treatment is likely to be more effective than CCBT or wait list given our results with social phobia which reflect this.

c) This study compares the intervention with the usual computerised CBT and with wait list thus controlling for placebo response and natural history. NB after 13 weeks wait list subjects are offered active intervention. The time on wait list is generally equivalent or less than what individuals would be waiting for any public sector treatment for generalised anxiety disorder.

d) Variations in responses will be treated individually through individual email contact during the program and further follow up and referral if required.

e) Endpoints:end of treatment questionnaires (1 week post treatment and 3 month follow–up).

f) Daily surveillance of patient progress, defined action: email, phone or GP contact if deterioration is suspected.

g) This is unlikely to be a consideration. Patients are welcome to withdraw at any time and seek alternative forms of help.

4. How many drugs will be used in this research project?

0

10. Declarations And Signatures

Applicant / Principal Researchers (including students where permitted)

Project Title (in full):	Distance treatment III: Randomised controlled trial in Generalized Anxiety Disorder
HREC to which this application is made:	St Vincent's Hospital Human Research Ethics Committee (EC00140)
HREC Reference number:	

I/we certify that:

- All information is truthful and as complete as possible.
- I/we have had access to and read the National Statement on Ethical Conduct in Research InvolvingHumans.
- The research will be conducted in accordance with the National Statement.
- The research will be conducted in accordance with the ethical and research arrangements of the organisations involved.
- The research will be conducted in accordance with the ethical and research arrangements of the organisations involved.
- I/we have consulted any relevant legislation and regulations, and the research will be conducted in accordance with these.
- I/we will immediately report to the HREC anything which might warrant review of the ethical approval of the proposal (NS 2.37), including:
- serious or unexpected adverse effects on participants;
- proposed changes in the protocol; and
- unforseen events that might affect continued ethical acceptability of the project.
- I/we will inform the HREC, giving reasons, if the research project is discontinued before the expected date of completion (NS 2.38);
- I/we will not continue the research if ethical approval is withdrawn and will comply with any special conditions required by the HREC (NS. 2.45);
- I/we will adhere to the conditions of approval stipulated by the HREC and will cooperate with HREC monitoring requirements. At a minimum annual progress reports and a final report will be provided to the HREC.

Applicant / Chief Researcher

Dr Nickolai Titov
UNSW at St Vincent's Hospital, Sydney

Signature: [signature] Date: 19/06/09

Principal Researchers

Professor John Gavin Andrews
UNSW at St Vincent's Hospital, Sydney

Signature: [signature] Date: 19/6/09

Associate Researchers

Dr Emma Robinson
St Vincent's Hospital, Sydney

Signature: [signature] Date: 19/6/09

Supervisor(s) of student(s)

Project Title (in full):	Distance treatment III: Randomised controlled trial in Generalized Anxiety Disorder
HREC to which this application is made:	St Vincent's Hospital Human Research Ethics Committee (EC00140)
HREC Reference number:	

I/we certify that:

- I/we will provide appropriate supervision to the student to ensure that the project is undertaken in accordance with the undertakings above;
- I/we will ensure that training is provided necessary to enable the project to be undertaken skilfully and ethically.

Heads of departments/schools/research organisation

Project Title (in full):	Distance treatment III: Randomised controlled trial in Generalized Anxiety Disorder
HREC to which this application is made:	St Vincent's Hospital Human Research Ethics Committee (EC00140)
HREC Reference number:	

I/we certify that:

- I/we are familiar with this project and endorse its undertaking;
- the resources required to undertake this project are available;
- the researchers have the skill and expertise to undertake this project appropriately or will undergo appropriate training as specified in this application.

Mr.
Title

Steven
First Name

Bernardi
Surname

A/Program Director
Position

SVHNS
Organisation Name

[signature]
Signature

22/6/09
Date

11. Attachments

List of Attachments

Core Attachments	Attachments which may be required/appropriate
Recruitment/invitation	Copy of advertisement, letter of invitation etc
Participant Information	Copy or script for participant<br>Copy or script for parent, legal guardian or person responsible as appropriate
Consent Form	Copy for participant<br>For parent, legal guardian or person responsible as appropriate<br>For, optional components of the project eg. genetic sub study
Peer review	Copy of peer review report or grant submission outcome
HREC approvals	Copy of outcome of other HREC reviews

Attachments specific to project or participant group	Attachments which may be required/appropriate
People whose primary language is other than English (LOTE)	English translation of participant information/consent forms

Participant information elements

Core Elements
Provision of information to participants about the following topics should be considered for all research projects.

Core Elements	Issues to consider in participant information
About the project	Full title and / or short title of the project<br>Plain language description of the project<br>Purpose / aim of the project and research methods as appropriate<br>Demands, risks, inconveniences, discomforts of participation in the project<br>Outcomes and benefits of the project<br>Project start, finish, duration
About the investigators / organisation	Researchers conducting the project (including whether student researchers are involved)<br>Organisations which are involved / responsible<br>Organistions which have given approvals<br>Relationship between researchers and particpants and organisations
Participant description	How and why participants are chosen<br>How participants are recruited<br>How many participants are to be recruited
Participant experience	What will happen to the particant, what will they have to do, what will they experience?

	Benefits to individual, community, and contribution to knowledge<br>Risks to individual, community<br>Consequences of participation
Participant options	Alternatives to participation<br>Whether participation may be for part of project or only for whole of project<br>Whether any of the following will be provided: counselling, post research follow–up, or post research access to services, equipment or goods
Participants rights and responsibilities	That participation is voluntary<br>That participants can withdraw, how to withdraw and what consequences may follow<br>Expectations on participants, consequences of non–compliance with the protocol<br>How to seek more information<br>How to raise a concern or make a complaint
Handling of information	How information will be accessed, collected, used, stored, and to whom data will be disclosed<br>Can participants withdraw their information, how, when<br>Confidentiality of information<br>Ownership of information<br>Subsequent use of information<br>Storage and disposal of information
Unlawful conduct	Whether researcher has any obligations to report unlawful conduct of participant
Financial issues	How the project is funded<br>Declaration of any duality of interests<br>Conspensation entitlements<br>Costs to participants<br>Payments, reimbursements to participants<br>Commercial application of results
Results	What will particpants be told, when and by whom<br>Will individual results be provided<br>What are the consequences of being told or not being told the results of research<br>How will results be reported / published<br>Ownership of intellectual property and commercial benefits
Cessation	Circumstances under which the participation of an individual might cease<br>Circumstances under which the project might be terminated

Research Specific Elements
Provision of information to participants about the following topics should be considered as may be relevant to the research project.

Specific to project or participant group	Additional issues to consider in participant information

St Vincent's Hospital

A facility of St Vincents
& Mater Health Sydney

St Vincent's Hospital Sydney Ltd
ABN 77 054 038 872
390 Victoria Street
Darlinghurst NSW 2010
Australia

T + 61 2 8382 1111
F + 61 2 9332 4142
www.stvincents.com.au

PARTICIPANT INFORMATION SHEET

Distance Treatment III: Randomized controlled trial in Generalised Anxiety Disorder (GAD).

Invitation

You are invited to participate in a research study into a distance treatment for GAD. We also refer to this as the Worry Program. The study is being conducted by Dr Nickolai Titov of the Anxiety Disorders Research Unit at St Vincent's Hospital, Sydney.

Before you decide whether or not you wish to participate in this study, it is important for you to understand why the research is being done and what it will involve. Please take the time to read the following information carefully and discuss it with others if you wish. We do know, that in order to have received this Participant Information Statement and Consent form you have read information on www.virtualclinic.org.au about the Worry Program. You have also completed the required questionnaires on this internet site that suggest you are eligible to participate in this study.

'What is the purpose of this study?'

The purpose is to investigate the feasibility of a new internet therapy for GAD. We have developed a new internet based program for adults with GAD. It comprises an illustrated story of someone recovering from GAD, take home tasks to facilitate your recovery, and for some participants, postings to a private internet forum to learn how others are progressing and regular clinician advice to improve progress. A username (your email address) and password is required for you to access this internet therapy.

This is a trial compares three groups: people with GAD who receive either 1) internet therapy plus weekly telephone reminders from the clinic manager, with 2) internet therapy plus help from an experienced clinician, with 3) being on a waitlist and not receiving treatment from us for up to 13 weeks.

We will recruit 120 people to the trial and randomly allocate people to one of the three conditions so that we can compare the three conditions. If you are allocated to the waitlist group we will be pleased to offer you active treatment after you complete another set of questionnaires at the 13 week point.

The treatment program will last up to 10 weeks. We will measure changes in symptom level over the course of treatment and compare progress of the treated groups with the group waiting for treatment. We will also measure

participation in lessons, homework and forum, and satisfaction with this mode of treatment.

'Why have I been invited to participate in this study?'
You are eligible to participate in this study because the investigators of this study have assessed the results of the questionnaires that you completed on www.virtualclinic.org.au and the results indicate that you could be suitable.

We will need you to email us back the consent form after we have telephoned you to confirm that you are suitable for the program and to answer any questions you might have. Each time you log on to do a lesson we will take that as a sign of continued consent to participate.

'What if I don't want to take part in this study, or if I want to withdraw later?'
Participation in this study is voluntary. It is completely up to you whether or not you participate. If you wish to withdraw from the study once it has started, you can do so at any time without having to give a reason. If you email us that you wish to withdraw, we will confirm the receipt of your email, and no further contact with you will occur.

'What does this study involve?'
We will telephone you to confirm your suitability by taking you through a diagnostic interview that will take some 10 to 15 minutes, and then we will answer any questions you may have. When we get your emailed consent we will register you as a patient of St Vincent's Hospital, Sydney. Because this is a research trial, you will be randomly allocated to 1) internet therapy with telephone reminders, 2) internet therapy with clinician help, or 3) waitlist. You will start treatment if you are allocated to either treatment groups, or some 13 weeks later if you are in the waitlist group.

The treatment program will be conducted online. As part of the study you will log on to www.virtualclinic.org.au at least every week during the program.

- Before you start the lessons you will need to fill in some diagnostic questionnaires.

You will read each of the six Lessons in the recovery story. Each lesson involves new educational material and homework tasks.

Lesson One:
- Learn about the physical, cognitive and behavioural symptoms of GAD.
- Learn about treatments for GAD.

Lesson Two:
- Begin planning pleasant activities.
- Learn about the relationship between the way you think and feel.

Lesson Three:
- Learn about changing thinking to improve your mood.
- Learn about the role of self-criticism in GAD.

Lesson Four:

- Learn about solving problems in a structured way.
- Learn about the role of avoidance in GAD.
- Design a plan for confronting problems, slowly and gradually.

Lesson Five:
- Learn about being assertive.
- Learn about improving your communication skills.

Lesson Six:
- Revise the skills you have learnt.
- Make a plan for staying well in the future

You will download the take home information and carry out the recommended exercises that aim to give you control over your GAD.

If you are in the Clinician guided group you will also be encouraged to participate in a secure and confidential online discussion forum with other people who are completing the program. You will post your thoughts about your progress with the homework. We strongly recommend you use a made up name, not your real name, when posting on the forum. One of the study clinicians will keep watch on your participation and offer comments in postings on the forum.

Your participation will end after a maximum of ten weeks, when you complete the second set of diagnostic questionnaires.

'How is this study being paid for?'
It is part of St Vincent's Hospital's Anxiety Disorders Clinic's services for people with GAD.

'Are there risks to me in taking part in this study?'
None that are known. If there are difficulties or complications you should contact the study clinician who will arrange appropriate help.

'Will I benefit from the study?'
This study aims to further medical knowledge and may improve future treatment of GAD. However it may or may not directly benefit you.

'Will taking part in this study cost me anything, and will I be paid?'
Participation in this study will not cost you anything other than the costs associated with using your computer or accessing the internet.

'How will my confidentiality be protected?'
All data concerning your identity, condition and progress will be lodged in our secure electronic database and in your medical record which is maintained by the hospital as a confidential document. Of the people treating you, only your clinician and the research staff will know whether or not you are participating in this study. Any identifiable information that is collected about you in connection with this study will remain confidential and will be disclosed only with your permission, or except as required by law. Only the researchers will

have access to your details which will be held securely at St. Vincent's Hospital.

'What happens with the results?'
We will analyse the results of your questionnaires and comments to allow us to improve the program. We plan to publish the results of your group in peer-reviewed journals, at presentations at conferences or in other professional forums. In any publication, information will be provided in such a way that you cannot be identified. Results may be discussed with the HREC for monitoring purposes.

'What happens to my treatment when the study is finished?'
You are being offered up to ten weeks of treatment. At the end of the program a Doctor from the Anxiety Disorders Clinic will discuss your progress and make suggestions as to how you might best manage your GAD in the future.

'Who should I contact if I have concerns about the conduct of this study?'
This study has been approved by St Vincent's Hospital HREC. Any person with concerns or complaints about the conduct of this study should contact the Executive Officer in the Research Office who is the person nominated to receive complaints from research participants. You should contact them on 02 8382 2075 and quote 08/SVH/36.

Thank you for taking the time to consider this study.

This information sheet is for you to keep.

We will send you an email consent document after the telephone interview. Only when you return this document will you be included in the study.

A facility of St Vincents
& Mater Health Sydney

St Vincent's Hospital Sydney Ltd
ABN 77 054 038 872
390 Victoria Street
Darlinghurst NSW 2010
Australia

T + 61 2 8382 1111
F + 61 2 9332 4142
www.stvincents.com.au

CONSENT FORM

Distance Treatment III: Randomised Controlled Trial of Generalized Anxiety Disorder

Please send this email back to the contact@virtualclinic.org.au by clicking the "reply" button.

When we receive your email we will regard you as having consented to join the study.

1. I would like to participate in this study

2. I acknowledge that I have read the participant information statement, which explains why I have been selected, the aims of the study and the nature of my participation and the possible risks and benefits.

3. I have been telephoned and given the opportunity of asking any questions about my participation and about any risks and benefits.

4. I understand that I can withdraw from the study at any time without prejudice to my relationship to St Vincent's Hospital, Sydney.

5. I agree that research data gathered from the results of the study may be published, provided that I cannot be identified.

6. I understand that if I have any questions relating to my participation in this research, I may contact Dr Nickolai Titov on telephone +612 8382 1732, who will be happy to answer them.

Complaints may be directed to the, Executive Officer, St Vincent's Hospital Research Ethics Committee DARLINGHURST 2010 AUSTRALIA (phone 8382 2075, fax 8382 3667, email research@stvincents.com.au).

Protocol Title

Distance treatment III: Randomised controlled trial in Generalized Anxiety Disorder

HREC Ref:

SVH Ref:

Version Number: 1

Date of Protocol: 17/06/09

SYNOPSIS

Protocol title: Distance treatment III: Randomised controlled trial in Generalized Anxiety Disorder

Protocol version: 1

LIST OF INVESTIGATORS

Chief Investigator: N Titov
Address: St Vincent's Hospital
Telephone no.: +612 8382 1732
Fax no.: +612 8382 1721

Principal Investigator: J G Andrews
Address: St Vincent's Hospital
Telephone no.: 8382 1726
Fax no.: 83821721

Associate Investigator: E Robinson
Address: St Vincent's Hospital
Telephone no: 8382 1729
Fax no.: 83821721

Summary

Protocol title:	Distance treatment III: Randomised controlled trial in Generalized Anxiety Disorder
Protocol version:	1
Objectives	Primary objective: To explore the outcomes of the clinician assisted computerized CBT (CaCCBT) programs for people with Generalized Anxiety Disorder (GAD) compared to the status of people who receive computerized CBT (CCBT) or who remain on the wait list using a randomized controlled trial design.<br>Secondary objectives: To further determine the feasibility of these treatments in terms of acceptability to patients and practicality for clinicians.
Study design:	A CONSORT compliant, registered RCT of the intervention (CaCCBT) versus CCBT versus waitlist control group.
Planned sample size:	120
Selection criteria:	Meets criteria for GAD as a primary diagnosis.
Study procedure	Recruit and treat subjects with GAD
Statistical considerations:	Sample size calculation: Assuming ES > 0.5<br>Analysis plan: ANCOVA
Duration of the Study:	12 weeks

TABLE OF CONTENT PAGE

1. BACKGROUND
   1.1. DISEASE BACKGROUND
   1.2. RATIONALE FOR PERFORMING THE STUDY
2. STUDY OBJECTIVES
   2.1. PRIMARY OBJECTIVE
   2.2. SECONDARY OBJECTIVES
3. STUDY DURATION AND STUDY EARLY TERMINATION
4. NUMBER OF CENTERS
5. SELECTION CRITERIA
   5.1. TOTAL NUMBER OF PATIENTS
   5.2. INCLUSION CRITERIA
   5.3. EXCLUSION CRITERIA
6 STUDY DESIGN
   6.1. DESIGN
7. STATISTICAL CONSIDERATIONS
   7.1. SAMPLE SIZE CALCULATION
   7.2. ANALYSIS PLAN
8 REFERENCES
9 APPENDICES

1. BACKGROUND:

1.1. DISEASE BACKGROUND

In the Australian National Survey of Mental Health and Well Being only 38% of people with Generalized Anxiety Disorder (GAD) reported seeing a physician and approximately only half got adequate treatment. (Andrews et al, 2004). The reason for the shortfall is well known (Andrews et al, 2001) being a mixture of societal, attitudinal and diagnostic variables. Making treatment more freely available in areas where expert treatment is not available, or to people who are unable to take time off work to access treatment is one logical step. This series of studies aims to achieve this by replicating a methodology which has been shown to be effective for the treatment of social phobia and depression and applying it to GAD.

GAD responds to cognitive behaviour therapy (CBT) and to medication with SSRIs (Nathan & Gorman, 2007). CBT for GAD and other anxiety disorders can be delivered over the internet, however, one difficulty with computerised CBT (CCBT) is that adherence is usually extremely poor in the absence of clinician input (Titov, 2007). We recently completed a randomised controlled trial of clinician assisted computerised CBT (CaCCBT) for the distance treatment of people with social phobia (Titov, Andrews, Schwencke, Drobny, & Einstein, 2008). Adherence was 80%. People in the intervention groups were as severe as people seen in the specialised anxiety disorders clinic at St Vincent's Hospital and yet made the same level of improvement as those receiving face to face treatment (ES respectively 0.8 clinic, 1.2 CaCCBT) but the latter group only required one quarter as much clinician time. This finding has been replicated (Titov, Andrews & Schwencke, 2008).

We are interested in whether similar results can be found for a population of people suffering from GAD.

1.2. RATIONALE FOR PERFORMING THE STUDY

Many people with GAD do not access treatment. We have developed an Internet based treatment program for GAD, which is currently being piloted in an RCT (treatment vs waitlist control). Preliminary results indicate that this treatment program is effective.

This proposed study will recruit 120 people with GAD and randomly allocate them to one of three groups: (1) Clinician guided internet-based treatment group, (2) Self-guided internet-treatment group, (3) Wait-list control group (who will receive treatment after the treatment groups have completed the program). We will measure changes in symptom level from the beginning of the 10 week program course of treatment and one week after the conclusion of treatment. Measurement in the control group will be matched to this. We will also measure adherence to the lessons, homework and forum, satisfaction with the clinician's input and satisfaction with the mode of treatment generally.

This proposed project employs the same research design as that previously approved by St Vincent's Hospital Human Research Ethics Committee ((EC00140)(AB/3097/1). However, 2 additional questionnaires have been added for assessing GAD at pre and post treatment.

2. STUDY OBJECTIVES

2.1. PRIMARY OBJECTIVES

To explore the outcomes of the clinician assisted CBT programs for people with GAD compared to the status of people who receive CCBT or who remain on the wait list.

2.2. SECONDARY OBJECTIVES

To further determine the feasibility of these treatments in terms of acceptability to patients and practicality for clinicians.

3. STUDY DURATION

1st September, 2009 to 1st December, 2009, 3 months

Participants will be encouraged to complete one lesson per week with the whole six lessons being completed within ten weeks. Adherence will be monitored on a daily basis via the course management system by the therapist, Dr Emma Robinson.

4. NUMBER OF CENTERS

1, St Vincent's Hospital only (120 patients)

5. SELECTION CRITERIA

5.1. TOTAL NUMBER OF PATIENTS

120 in total.

CaCCBT: n=40

CCBT: n = 40

waitlist: n= 40

5.2. INCLUSION CRITERIA/EXCLUSION CRITERIA:

The criteria for diagnosis of GAD will be based on the Mini International Neuropsychiatric Interview (Appendix A).

Aged over 18, self identified as suffering from GAD and have questionnaire scores and results of telephone diagnostic interview consistent with this; no history of psychosis or current alcohol and drug dependence, not currently suicidal; have access to phone and computer with printer. Prepared to provide name, phone number and address and to be registered as a patient of St Vincent's Hospital, Sydney, and to provide the name and address of a local general practitioner and to provide written informed consent

6. STUDY DESIGN

6.1. STUDY DESIGN:

A CONSORT compliant, registered RCT of the intervention (CaCCBT) versus CCBT versus waitlist control group. This will demonstrate whether the benefit from the CaCCBT intervention is superior to natural remission and placebo response. Because of low adherence to CCBT we think that it is a proxy for a placebo treatment. As in any RCT accurate patient selection, good randomization, reliable and valid outcome measures, and low drop out rates are critical. Analyses based on completers and on intention to treat will be performed.

There are already 200 people on the waitlist for virtual clinic. In addition, some media attention is expected to generate more publicitiy to attract people with GAD to the program. The study will be described on the intake website www.virtualclinic.org.au. People will read the information about the studies and elect to continue or not. If they choose to apply for the study they will complete an

automated screening questionnaire. Only people whose responses to the questions meet selection criteria and undergo a brief phone interview explaining the program will read the information sheet and return an electronic consent form and mail that to the investigators. This will trigger the phone interview in which the diagnosis will be confirmed using the Mini International Neuropsychiatric Interview (Appendix A). Questions about the study will be answered and an offer of treatment made.

Patients with GAD will be randomly allocated to CaCCBT group (n=40), CCBT group (n=40), or the waitlist group (n=40. Randomization will be done via www.random.org.

Upon beginning treatment, all participants will complete a set of diagnostic questionnaires. The questionnaires being administered in this study pre and post treatment are the same as the previously approved study by St Vincent's Hospital Human Research Ethics Committee ((EC00140)(AB/3097/1). There are two additional questionnaires. These are the GAD-7 (Short measure of Generalised Anxiety Disorder. Spitzer R, Kroenke, K, Williams J, Löwe, B(2006)) and the PSWQ: Penn State Worry Questionnaire (Meyer et al, 1990)

For the treatment groups, Dr Emma Robinson will treat the participants. The CaCCBT treatment is delivered via the website www.virtualclinic.org.au. The treatment program consists of 6 lessons conducted over a 10 week period, in which participants are encouraged to complete one lesson per week. Participants in the CaCCBT group will receive regular email contact with their clinician, and will be encouraged to carry out homework activities and participate in a secure online forum.

The patients in the CCBT groups will have same access to the lessons, homework activities as the CaCCBT groups, and a secure online forum for their own CCBT group. Unlike the CaCCBT groups, the CCBT groups will have no clinician contact over the 10 week period. The waitlist group will not receive any treatment during this period.

After the treatment group has finished the program, all participants will complete a second set of diagnostic questionnaires. The waitlist and CCBT group will be offered effective treatment after this.

Effectiveness of the CaCCBT program for GAD will be measured by comparing waitlist and CCCBT and CaCCBT groups on their pre- and post- treatment scores.

7. STATISTICAL CONSIDERATIONS

7.1. Sample Size Calculation

We would expect pre-post improvement of ES 1.0 for the CaCCBT groups on Generalised Anxiety Disorder measures. We also expect the CaCCBT group to improve more than the CCBT group by an ES of 0.3 and waitlist group by an ES of 0.6. Sample size is powered to have an 80% chance of detecting differences at $p<.05$.

7.2. Analysis Plan

Analyses based on completers and on intention to treat will be performed. Three mixed design ANCOVAs will be carried out utilising SPSS GLM with the baseline score entered as a covariate. The within subject factor examines the difference in pre-post scores for each of the three treatment conditions, whilst the between subjects factor compares improvement scores between treatment groups.

8. REFERENCES

Andrews, G., Henderson, S., & Hall, W. (2001). Prevalence, comorbidity, disability and service utilization. Overview of the Australian Mental Health Survey. *British Journal of Psychiatry,178*, 145-153.

Andrews G., Issakidis, C., Sanderson, K., Corry, J., & Lapsley, H. (2004). Utilising survey data to inform public policy: comparison of the cost-effectiveness of treatment of ten mental disorders. *British Journal of Psychiatry, 184*, 526-533.

Nathan, P. E. & Gorman, J. M. (Ed) (2007). *A guide to treatments that work (3rd ed.)*. Oxford University Press: NY

Meyer, T., Miller, M., Metzger, R & Borkovec, T (1990). Development and validation of the Penn State Worry Questionnaire. *Behaviour Research and Therapy*, 28, 487-495.

Spitzer R, Kroenke, K, Williams J, Löwe, B (2006)) A brief measure for assessing Generalised Anxiety Disorder GAD-7. Archives of internal medicine, 166(10), 1092-1097.

Titov, N. (2007). Status of computerized cognitive behavioural therapy for adults. *Australian and New Zealand Journal of Psychiatry, 41*, 95-114.

Titov, N., Andrews, G., & Schwencke, G. (2008). Shyness 2: Treating social phobia online. Are the results reliable, and who benefits? Australian and New Zealand Journal of Psychiatry.

Titov, N., Andrews, G., Schwencke, G., Drobny, J., & Einstein, D. (2008). "Shyness 1", a randomized controlled trial of an Internet-based treatment for social phobia. Australian and New Zealand Journal of Psychiatry.
